# Bee Venom Acupuncture Effects on Pain and Its Mechanisms: An Updated Review

**DOI:** 10.3390/toxins13090608

**Published:** 2021-08-29

**Authors:** Soo-Hyun Sung, Gihyun Lee

**Affiliations:** 1 Department of Policy Development, National Development Institute of Korean Medicine, Seoul 04554, Korea; koyote10010@nikom.or.kr; 2 College of Korean Medicine, Dongshin University, Naju 58245, Korea

**Keywords:** bee venom, bee venom acupuncture, musculoskeletal pain, inflammatory pain, neuropathic pain, traditional, complementary and alternative medicine

## Abstract

Bee venom (BV) is a complex natural toxin that contains various pharmaceutical compounds. Bee venom acupuncture (BVA), involving a BV injection into a certain acupuncture point, has been utilized to relieve a range of pain conditions. Regardless of whether pain is caused by disease or injury, if not effectively treated, pain can exert a detrimental effect on all aspects of life. In the past decade, many researchers have investigated the anti-nociceptive effects of BVA through clinical use and experimental evaluation. This report reviews the existing knowledge on the analgesic effects of BVA, focusing on musculoskeletal pain, inflammatory pain and neuropathic pain, and its analgesic mechanisms. Although further clinical trials are needed to clinical application of experimental results, this review will contribute to the standardization and generalization of BVA.

## 1. Introduction

Pain is a worldwide health problem and is highly prevalent [1,2]. The prevalence of pain is estimated at 20% [3,4,5]. Depending on the cause, such as diseases or injury, pain intensity varies from mild to severe [6,7]. Regardless of the type, untreated pain can affect the quality of life and may lead to psychological, physical, and social consequences [8,9]. 

Bee venom (BV) is an animal toxin that contains several active compounds, such as enzymes, peptides, non-peptide components, and biologically active amines. BV demonstrates the potential to be used as a beneficial pain treatment [10,11]. There are several types of BV therapies available, such as bee sting therapy, bee venom acupuncture (BVA), and the external use of BV products. Bee sting therapy involves the injection of BV through live bee stings on human skin. For this reason, this treatment modality also exhibits a high risk of developing adverse side effects [12]. On the other hand, BVA, which involves the administration of BV diluted with saline into a specific acupoint, has been used to treat different types of pain in the clinical fields of traditional, complementary, and alternative medicine [10] and does not exhibit the same adverse risks as those observed with bee sting therapy.

In previous studies, Chen et al. [13] reviewed papers published before 2010 regarding the pain reduction effects of BV injection, and its analgesic and anti-nociceptive mechanisms. They highlighted that therapeutic BV injection might be beneficial for certain patients but may also involve risks. In this review, we summarise information published over a decade of research conducted on the potential therapeutic applications and mechanisms of BVA for pain treatment. Thus far, painful conditions where therapeutic applications of BVA have been studied include musculoskeletal pain (e.g., shoulder pain and low back pain), inflammatory pain (e.g., osteoarthritis pain and arthritis pain), neuropathic pain (e.g., post-stroke pain, complex regional pain syndrome, refractory postherpetic neuralgia, mechanical allodynia, cold allodynia, and intervertebral disc disease-induced neurological pain), post-ischaemic pain, and prostatic pain. The summary is provided preceding the discussion section of our review, and in the order of clinical studies and experimental studies (Table 1, Table 2, Table 3, Table 4, Table 5 and Table 6).

## 2. Summary of the Previous Review

Chen et al. [13] collected and reviewed articles on the nociceptive and anti-nociceptive effects of BV therapy published before 2010. The main components of BV are peptide constituents such as enzymes (e.g., phospholipase A2 (PLA_2_) and hyaluronidase), apamin, melittin, mast cell degranulating peptide, and adolapin. Among them, melittin (40–60%), adolescent (1%), and PLA_2_ (12%) were reported to exert antinociceptive effects. Mast-cell degranulating peptide (2–3%) and hyaluronidase (<3%) produced histamines associated with allergic reactions to BV therapies. Experimental human and animal studies conducted on BV-induced pain, inflammation, and allergic responses were examined. Honeybee stings cause hyperalgesia, long-lasting pain, and local inflammation, suggesting that live bee sting therapy may cause such adverse events. Additionally, a significant number of papers have been conducted in the past decade, highlighting the anti-inflammatory and anti-nociceptive effects of BV therapies and components of BV. In clinical evidence, the effects of BVA for treating pain (e.g., pain of osteoarthritis of the knee, and musculoskeletal pain) have been reported. The anti-inflammatory and anti-nociceptive effects of BVA have been demonstrated more comprehensively in various animal pain models, including those pertaining to the study of adjuvants (rheumatoid arthritis model), collagen type-II (inflammatory model), carrageenan (inflammatory model), and chronic constriction injury (neuropathic pain model).

## 3. Therapeutic Effects of BVA in Pain

### 3.1. Musculoskeletal Pain

#### 3.1.1. Clinical Studies

##### Shoulder Pain

In a systematic review involving seven randomized controlled trails (RCTs) [14], shoulder pain was observed to be significantly lower for BVA (administration route (AR): GB21, LI11, LI15, LI16, SI3, SI9, SI10, SI11, TE14, and UE12 acupoints) plus conventional treatment (CT) than that reported for saline injection plus CT on the pain rating scale (PRS) (*p* = 0.009) and visual analogue scale (VAS) (*p* = 0.03). The BVA concentrations of the collected RCTs ranged from 0.03–0.5 mg/mL, the total treatment sessions were 6–16 times, and the total volume ranged from 0.6–14.8 mL. Among the 45 patients who underwent BVA, 2 presented with pain, 8 exhibited pruritus, 3 experienced a burning sensation, 30 presented with pruritus/local swelling/redness, and 1 showed mild, generalised swelling/aching. This study suggests that shoulder pain can be treated considering the BVA procedure in addition to CT, but standardisation of the dose and the total number of procedures should be considered.

In an RCT reported by An et al. [15], a total of 101 patients received either conventional or needle-free BVA for examination of the therapeutic effects of BVA on myofascial shoulder pain. BV was administered to the GB21 acupoint for both groups, and shoulder pain was significantly improved by the treatment in both groups. The concentration of BV was not reported, and a total of two treatment sessions were performed, with a total volume of 2 mL. Adverse events (AEs) were less frequently reported in the needle-free group (inflammation, 1; purpura, 2; headache, 2; and redness, 1) than in the BVA (fatigue, 1; purpura, 1; oedema, 2; headache, 1; cold, 1; itching, 16; and redness, 14) groups.

Two RCTs [16,17] reported the assessment of BVA for adhesive capsulitis pain. One study [16] recruited 68 patients into three groups: BVA group1 (0.1 mg/mL BVA plus physiotherapy), BVA group2 (0.03 mg/mL BVA plus physiotherapy), and control group (saline injection plus physiotherapy). Both BVA groups injected BV into GB21, LI15, LI16, SI11, and TE14 acupoints. Compared to the control group, the BVA group1 presented significant improvements in the shoulder pain and disability index (SPADI) at 8 days (*p* = 0.025) and 12 days (*p* = 0.014), and VAS score at rest at the eighth week (*p* = 0.048), during the motion score at the 12th week (*p* = 0.029). Both BVA groups received 16 sessions of treatment, and the total BV volume was 14.8 mL. A one-year follow-up analysis of a previously reported RCT [17] was conducted using a tele-interview. The SPADI scores at one-year statistically differed between the 0.1 mg/mL BVA plus physiotherapy group and the saline injection plus physiotherapy group (*p* = 0.043). The therapeutic effect on adhesive capsulitis pain was partially confirmed using BVA at a concentration of 0.1 mg/mL rather than at a concentration of 0.03 mg/mL, and minor AEs were confirmed.

##### Low Back Pain

Two RCTs [18,19] and one retrospective study [20] examined the treatment effect of BVA on low back pain. In a RCT reported by Seo et al. [18], a total of 54 subjects received either BVA (AR: BL23, BL24, BL25, GB30, GV3, GV4, and GV5 acupoints) plus non-steroidal anti-inflammatory drugs (NSAIDs) or saline injection plus NSAID for six treatments over a period of 3 weeks. The BVA plus NSAID group showed a significant reduction in chronic low back pain (*p* = 0.0486) compared to the control group, as measured by using VAS. The BVA concentration was 0.05 mg/mL, and the total volume was 28 mL across six treatment sessions. Among the 27 subjects treated with BVA, itching/sensation was observed in four patients, headache occurred in one patient, and generalised myalgia occurred in one patient. Shin et al. [19] randomly divided 60 patients into BVA (AR: BL23, BL24, and BL25 acupoints) and saline injection groups. In VAS used for pain intensity, the BVA group showed a statistically significant difference (*p* = 0.0087) compared to saline injection. The BVA concentration was 0.05 mg/mL, the total treatment sessions were eight times, and the total volume was 4.8 mL. Among the 30 subjects treated with BVA, 15 presented with pruritus, 5 experienced skin flares, 4 showed oedema, and 2 presented with skin rash.

In a retrospective observational study of BVA (AR: 4–5 acupoints around the lumbar spine) for low back pain of intervertebral disc herniation [20], a total of 524 participants who received non-surgical complementary and alternative medicine treatment (BVA, acupuncture, herbal medicine, and chuna) exhibited a numeral rating scale (NRS) score of 3.18–2.29 (95% confidence interval [CI], 2.99–3.38). The BVA concentration was 0.1 mg/mL, the total treatment sessions were 2.3 ± 1.8 times, and the total volume was not reported. Allergic reactions caused by BV were observed in eight patients. The clinical trial of Shin et al. [20] is not a single treatment of BVA but suggests that BVA is clinically used for low back pain. Additionally, in the two RCTs [18,19], the BVA concentration was the same at 0.05 mg/mL, but the total volume was approximately five times different. Standardisation of treatment sessions and dosage is required.

### 3.2. Inflammatory Pain

#### 3.2.1. Clinical Studies

BVA was used in 361 patients with knee osteoarthritis pain [21], and subjects were randomly assigned to the BVA or histamine injection groups. The BVA group injected BV into BL40, BL19, BL 21, BL 23, BL 25, BL27, ST34, five on each knee (knee top), eye—one medial, and eye—two lateral. The control group received the same acupoint injection as the BVA group. The BVA group showed significant improvement in the Western Ontario and McMaster Universities Osteoarthritis index (WOMAC) pain score after 12 weeks (95% CI, 0.3–2.0, *p* = 0.0010). BV powder 1 mg and 1 mL of 0.5% lidocaine were mixed, and 12 treatment sessions were conducted with a total dosage of 17.1 mL. Among the patients treated with BVA, AEs occurred in 177 patients and injection site AEs occurred in 15 patients.

#### 3.2.2. Experimental Studies

Two experimental studies [22,23] have investigated arthritis pain in a mouse model. BV (1 mg/200 g) showed anti-nociceptive activity in Freund’s complete adjuvant (FCA) and collagen type-II-induced rats with arthritis pain [22]. For FCA-induced rats, the dorsal flexion test score was significantly reduced in BVA (AR: right hind paw, dose: 1 mg/200 g) and conventional drug (indomethacin, 2 mg/kg) administration in comparison with the saline injection group. In the collagen type-II-induced rat model, BVA and indomethacin administered groups revealed a decrease in the dorsal flexion score compared to the saline-injected group. Yamasaki et al. [23] compared the effectiveness of BVA (AR: dorsal route, dose: 0.25 mg/kg in a volume of 50 µL) and methotrexate (AR: dorsal route, dose: 0.3 mg/kg in a volume of 300 µL), the most commonly utilized disease-modifying anti-rheumatic drug in rats with collagen type-II-induced arthritis pain. The value of the mechanical threshold of hyperalgesia in healthy rats was 46 ± 2. The reduced mechanical threshold of hyperalgesia persisted in all collagen type-II-induced rat groups, but with amelioration in relation to untreated arthritic rats (11 ± 1.30), in the methotrexate administration group (22 ± 1.70), and in the group subjected to a combination of BVA and methotrexate administration (23.5 ± 2.20).

Two experimental studies [24,25] have focused on formalin-induced inflammatory pain. Kang et al. [24] assessed the anti-nociceptive effect of BVA in ST36 acupoint in formalin-induced mice with inflammatory pain during the first and second round of the formalin test. Although injection of a low dose of 0.08 mg/kg BVA did not improve formalin-induced pain behaviour, a higher dose of 0.8 mg/kg BVA significantly improved pain responses in the second round of the formalin test compared to that of the saline injection group. Experiments were performed for assessing formalin-induced inflammatory pain responses elicited during the early and late round of the formalin test [25]. The results of BVA (AR: ST36 acupoint, dose: 0.8 mg/kg) and BVA plus hydroxydopamine (AR: intraperitoneal route, volume: 100 µL) showed significant improvements on formalin-induced pain behaviour as compared with the saline injection group during late round of the formalin test.

Huh et al. [26] investigated the analgesic effects of BVA in a rat model of collagen type-II-induced osteoarthritis pain. The BVA (AR: ST36 acupoint, dose: 1.0 mg/kg) resulted in a more significant pain reduction effect than the non-acupoint BVA (AR: intraperitoneal route, dose: 1.0 mg/kg) and no treatment groups. Pain-related response was more improved by treatment with 1 mg/kg BVA (AR: ST36 acupoint) than that observed with 2 mg/kg BVA (AR: ST36 acupoint).

### 3.3. Neuropathic Pain

#### 3.3.1. Clinical Studies

##### Post-Stroke Pain

A meta-analysis of two RCTs [27] showed that post-stroke shoulder pain was significantly lower in the BVA group (AR: EX-UE70, GB21, LI11, LI15, SI3, SI9, SI10, SI11, and TE14 acupoints) than that in the saline injection group in VAS (*p* = 0.02). The BVA concentration was 0.01–0.5 mg/mL, the total treatment sessions were 6–12 times, and the total volume was 0.9–13.5 mL. A single-blind RCT [28] investigated the central post-stroke pain. BVA or saline was administered into GB21, GB31, GB39, LI11, LI15, and ST36 acupoints. After three weeks, the VAS score improved more in the BVA group than that in the control group (*p* = 0.009). The concentration of BV was not reported, but the total volume was 1.8 mL over six treatment sessions. No AEs occurred in the BVA group. These studies indicate that BVA is effective for treating post-stroke pain and musculoskeletal pain.

##### Chemotherapy-Induced Peripheral Neuropathic Pain

Two case studies [29,30] focused on BVA for chemotherapy-induced peripheral neuropathic (CIPN) pain. Park et al. [29] treated four CIPN pain patients with BVA (0.1 mg/mL, three sessions of 4.8 mL) on Ba Xie and Ba Feng acupoints (total of 16 points located between all 5 fingers and toes). The average VAS score decreased from 8.75 to 2.75, and AEs were not observed. Yoon et al. [30] treated 11 patients with CIPN pain using BVA (0.1 mg/mL) on GB39, LI4, LV3, and SJ5 acupoints. The mean VAS score significantly decreased 3 weeks after the final treatment (*p* < 0.01) and continued to decrease after 6 weeks (*p* < 0.05). Minor AEs (swelling and itchiness, 2; mild fever, 1) were reported in the study. Regarding pain reduction mechanisms, Yoon et al. [30] reported that BVA may demonstrate functions based on mechanisms similar to those of adrenergic reuptake inhibitors or norepinephrine reuptake inhibitors. Two studies were conducted at the East-West Cancer Center of Dunsan Oriental Hospital of Daejeon University using the same concentration, dose and volume. This study will be developed as a dose–response evaluation to further establish the safety and response prior to clinical trial.

##### Complex Regional Pain Syndrome and Refractory Postherpetic Neuralgia

One patient diagnosed with complex regional pain syndrome after toe surgery received BVA at the GB43 acupoint [31]. After treatment, the worst level of the numeral rating scale (NRS) was reduced from 8 to 0, the usual level of NRS was reduced from 5 to 0, and the best level of NRS was reduced from 3 to 0. BVA concentration was not reported, the total number of treatment sessions was 14, and the volume was 4.55 mL. No side effects related to BVA occurred. In another study [32], one patient with refractory postherpetic neuralgia was treated with BVA (0.03 mg/mL) once a week for 4 weeks. The patient’s NRS score decreased from 8 to 2, and itchiness at the site of injection occurred for 2 days following treatment.

#### 3.3.2. Experimental Studies

##### Mechanical Allodynia

Many studies have studied whether BVA could be used to reduce mechanical allodynia, a representative symptom of neuropathic pain. Two experimental studies [33,34] investigated the use of BVA in spinal nerve ligation (SNL)-induced mechanical allodynia. In a study reported by Woo et al. [33], SNL was conducted in rats to induce neuropathic pain. SNL-induced mechanical allodynia rats were injected in the intraperitoneal route with 0.2 mg/kg PLA_2_ or 0.2 mg/kg phosphate-buffered saline (PBS). PLA_2_ is a main component of BV, consisting of 10%–12% of dry BV [54,55]. The PLA_2_ group showed a significant decrease in mechanical allodynia at 2 days (*p* < 0.001) compared to the control group. Perineural pre-treatment of the L5 and L6 spinal nerves with BVA significantly improved mechanical allodynia in SNL-induced rats [34]. The mechanical hind paw withdrawal threshold was more improved in rats treated with 0.05 mg/kg and 0.1 mg/kg BVA on days 3, 5, 7, 9, and 13 compared to the saline treated control group.

Two animal studies [35,36] focused on the effectiveness of BVA on paclitaxel-induced mechanical allodynia. A mechanical allodynia model was induced in rats treated with paclitaxel, a chemotherapy drug for various tumours [35]. Paclitaxel-induced rats were injected with 1.0 mg/kg BVA or PBS at the right side of the ST6 acupoint. The BVA group showed a significant effect in mechanical allodynia at 1 h (*p* < 0.05) and 2 h (*p* < 0.01) after injection. Li et al. [36] investigated the combined effects of BVA (AR: ST36 acupoint, dose: 1.0 mg/kg, BV dissolved in a volume of 20 μL PBS) and venlafaxine (AR: oral route, dose: 40 mg/kg, venlafaxine dissolved in a saline) on mechanical allodynia induced by paclitaxel, a primary chemotherapeutic agent utilized to treat various solid malignancies. Co-treatment with BVA and venlafaxine produced a longer-lasting anti-allodynic effect (180 min) than that induced by BVA (effects continued for 120 min) or venlafaxine (effects continued for 60 min).

Several studies [37,38,39,40,41,42,43] have examined the anti-allodynic effect of BVA on oxaliplatin-induced mechanical allodynia. Kim et al. [37] assigned 32 oxaliplatin-induced mechanical allodynia model mice into four groups. Oxaliplatin is a chemotherapeutic drug used to treat colorectal cancer that causes severe peripheral neuropathy. Both morphine (AR: intraperitoneal route, dose: 2.0 mg/kg in a volume of 0.2 mL) with a saline injection and BVA (AR: ST36 acupoint, dose: 1.0 mg/kg was injected in a volume 20 μL) with a saline injection exerted significant effects that continued 120 min after the treatment. However, these analgesic effects were less significant than the effect of the combination of BVA and morphine, which lasted 180 min. These results indicate that combination treatment of BVA and morphine is effective in oxaliplatin-induced mechanical allodynia. Li et al. [38] investigated the effects of BV (AR: not reported, dose: 1.0 mg/kg, BV dissolved in a PBS) and PLA_2_ (AR: not reported, dose: 0.2 mg/kg, PLA_2_ dissolved in a PBS) on oxaliplatin-induced mechanical allodynia in mice. BVA significantly reduced mechanical allodynia from day 2 and the effect continued until day 4. The PLA_2_ mice group showed a significant improvement in mechanical allodynia from day 2 and the effects lasted up to day 5. In the study reported by Li et al. [39], PLA_2_ (AR: intraperitoneal route, dose: 0.2 mg/kg) pre-treatment significantly improved mechanical allodynia on day 3. These results suggest that PLA_2_ has the potential to be used in the prevention of oxaliplatin-induced mechanical allodynia. Yoon et al. [40] evaluated oxaliplatin-induced mechanical allodynia in the hind paws of mice and examined the effect of BVA on mechanical allodynia. No significant differences were observed between the paw withdrawal threshold values before and after injection in the saline (AR: ST36 acupoint, dose: not reported) and the BVA-Back (AR: back point, dose: 0.1 mg/kg, BV dissolved in a volume of 20 μL saline) groups in hind paws. One hour after BVA (AR: ST36 acupoint, dose: 0.1 mg/kg, BV dissolved in a volume of 20 μL saline) treatment, significant increases in paw withdrawal threshold values were observed in the right hind paw compared with the saline injection group. Yeo et al. [41] investigated BVA (AR: ST36 acupoint, dose: 0.1 mg/kg, BV dissolved in a volume of 20 μL saline) in oxaliplatin-induced mice with mechanical allodynia. The results showed a significant long-lasting analgesic effect of repetitive 0.1 mg/kg BVA on the paw withdrawal threshold before and after subjection to BVA compared with the saline injection group (AR: ST36 acupoint, dose: not reported). In contrast, no significant differences were shown in the paw withdrawal threshold before and after injection in the non-acupoint BVA group (AR: back point, dose: 0.1 mg/kg, BV dissolved in a volume of 20 μL saline). Choi et al. [42] investigated the anti-nociceptive effect of melittin on oxaliplatin-induced mechanical allodynia. The mechanical allodynia was significantly reduced 30 min after melittin injection (0.5 mg/kg) on ST36 acupoint. In a study reported by Lee et al. [43], single subcutaneous administration of BVA (0.1 mg/kg and 1 mg/kg) at ST36 acupoint reduced mechanical allodynia induced by oxaliplatin in rats. Mechanical allodynia evaluated using von Frey tests was significantly improved 30 min after injection with 0.1 mg/kg and 1 mg/kg of BV compared to the control (post-injection; *p* < 0.01 and *p* < 0.001 for 0.1 mg/kg BV and 1 mg/kg BV, respectively).

Three animal studies [44,45,46] investigated a neuropathic pain rat model induced by chronic constrictive injury (CCI), spinal cord injury (SCI), and vincristine. In the study reported by Kang et al. [44], BV (AR: ST36 acupoint, dose: 0.25 mg/kg, BV dissolved in a volume of 50 μL saline) was injected subcutaneously twice a day for 2 weeks from 15 days after CCI surgery. Repeated administration of BV significantly reduced the paw withdrawal frequency from 4 days and continued for 13 days compared with the saline injection group (AR: ST36 acupoint, dose: not reported). Kang et al. [45] examined the effect of repetitive BVA (0.25 mg/kg) on SCI-induced mechanical allodynia. Repetitive BVA (AR: ST36 acupoint, dose: 0.25 mg/kg, BV dissolved in a volume of 50 μL saline) significantly increased in the paw withdrawal threshold in the ipsilateral hind paw on day 3 and 5 after treatment. Peripheral neuropathy was induced in rats with vincristine, a chemotherapy drug for solid and hematologic cancers [46]. 1.0 mg/kg BVA, 0.5 mg/kg melittin, 0.12 mg/kg PLA_2_, or saline was subcutaneously injected at the ST36 acupoint. Compared with the saline group, paw withdrawal was significantly decreased after BVA (1 mg/kg), which lasted until 60 min. The effect of melittin injection was significant and continued for 120 min. PLA_2_ injection showed no significant effect after treatment.

##### Cold Allodynia

Most animal studies [37,38,39,42,43,47,48,49] have examined the analgesic effect of BVA on oxaliplatin-induced cold allodynia. In a study reported by Kim et al. [37], a cold allodynia model was induced in mice treated with oxaliplatin, a chemotherapeutic drug for cancer. BVA (1.0 mg/kg was injected into ST36 acupoint in a volume 20 μL) with saline injection (AR: ST36 acupoint, volume: 0.2 mL) exerted a significant effect at the beginning (60 min), but its anti-allodynic effect could not be observed at 180 min after injection. Morphine (AR: intraperitoneal route, dose: 2.0 mg/kg in a volume of 0.2 mL) with saline injection (AR: intraperitoneal route, volume: 0.2 mL) did not exert any significant analgesic effect. The combination of BVA and morphine showed a significant and longer effect than that observed with treatment using either BVA or morphine alone. The cold allodynia model was induced in male mice treated with oxaliplatin [38]. BVA (AR: not reported, dose: 1.0 mg/kg, BV dissolved in a PBS) for five consecutive days significantly improved cold allodynia from day 3 and the effect continued up to day 5. PLA_2_ injection (AR: not reported, dose: 0.2 mg/kg, PLA_2_ dissolved in a PBS) significantly reduced cold allodynia from day 1, and this effect persisted for at least six days. These results indicate that PLA_2_ injection significantly alleviates oxaliplatin-induced cold allodynia. Li et al. [39] investigated the preventive effects of PLA_2_ on oxaliplatin-induced cold allodynia. Pre-treatment with PLA_2_ (AR: intraperitoneal route, dose: 0.2 mg/kg) once a day for five days significantly improved cold allodynia from days 3 to 7. Lee et al. [47] evaluated the effect of 5-HT depletion (AR: intraperitoneal route, dose 150 mg/kg) on the reduction effect of BVA (AR: GV3 acupoint, dose: 0.25 mg/kg, BV dissolved in a volume of 0.05 cc saline) on cold allodynia in oxaliplatin-treated mice. The control group (0.05 cc saline pre-treatment on GV3 acupoint) showed a significant improvement in tail withdrawal latency after 0.25 mg/kg BVA (*p* < 0.001), whereas the depletion of 5-HT by DL-pchlorophenylalanine (150 mg/kg) pre-treatment blocked the anti-allodynic effect of 0.25 mg/kg BVA (*p* > 0.05). These results indicate that the serotonergic system plays a main role in mediating the anti-nociceptive effect of BVA on oxaliplatin-induced cold allodynia. The melittin injection on ST36 acupoint reduced cold allodynia caused by oxaliplatin injection [42]. Cold allodynia was significantly improved after melittin injection (0.5 mg/kg). In contrast, saline injection (AR: ST36 acupoint, dose: not reported) did not exert any measurable effects on cold allodynia. Lee et al. [43] assessed the effects of 0.1 mg/kg BVA, 1.0 mg/kg BVA, or PBS, subcutaneously injected at the ST36 acupoint on oxaliplatin-induced cold allodynia model rats. The results showed that only BVA (1 mg/kg) showed a significant analgesic effect (*p* < 0.05) for cold allodynia, 30 min after BV injection. Yoon et al. [48] showed the relieving effect of BVA on oxaliplatin-induced cold allodynia. Behavioural tests for cold allodynia were conducted before oxaliplatin injection, 30 min after injection, and 60 min after treatment. The significant relieving effect of BVA (AR: GV3 acupoint, dose: 0.25 mg/kg, BV dissolved in saline at concentrations of 0.025 mg/mL) on cold allodynia was blocked by pre-treatment with 2 mg/kg mecamylamine (a non-selective nicotinic receptor antagonist), but not by 1 mg/kg atropine (a non-selective muscarinic receptor antagonist). Lim et al. [49] investigated whether BVA relieved oxaliplatin-induced cold allodynia. BVA (1.0 mg/kg) at GV3, LI11, and ST36 acupoints significantly improved cold allodynia; the anti-allodynic effect lasted for 2 h in the GV3 group and persisted for 1 h in the other groups (LI11 and ST36 acupoints).

In a study reported by Koh et al. [34], a cold allodynia model was induced in male rats with SNL. The cold allodynia in the both 0.1 mg/kg and 0.05 mg/kg BVA groups (AR: peri-neural sheath of the L5 and L6 spinal nerves) was observed on the first day of the acetone test and effects lasted until the completion of the study, with effects continuing for more than 29 days. The 0.1 mg/kg BV group showed effects that were significantly longer in the cold plate test compared with the 0.05 mg/kg BV group throughout the study duration.

Three animal studies [36,46,50] investigated a neuropathic pain mouse model induced by paclitaxel, vincristine, and chronic constriction injury (CCI). Li et al. [36] assessed the analgesic effects of paclitaxel on cold allodynia. The co-administration of BVA (AR: ST36 acupoint, dose: 1.0 mg/kg, BV dissolved in a volume of 20 μL PBS) and venlafaxine (AR: oral route, dose: 40 mg/kg, venlafaxine dissolved in a saline) significantly reduced licking and shaking frequencies (cold allodynia) compared to the BVA group from 60 min, and effects lasted up to 180 min. Li et al. [46] examined the effects of BVA in alleviating peripheral neuropathic pain induced in rats with vincristine. Behavioural tests were conducted for the ipsilateral hind paw before 1.0 mg/kg BVA, 0.5 mg/kg melittin, 0.12 mg/kg PLA_2_, or saline injection on the ST36 acupoint and were assessed at 30, 60, and 120 min after injection. Among the injected components, BVA significantly decreased cold allodynia in the acetone test, and the effects lasted up to 60 min. Kang et al. [50] investigated whether 0.25 mg/kg BVA or 2.5 mg/kg BVA reduced cold allodynia in sciatic nerve CCI rats. The single-high-dose BVA (2.5 mg/kg, twice a day for 2 weeks) on ST36 acupoint administered after CCI significantly improved the CCI-induced paw withdrawal frequency increase from 45 min to 90 min compared to the saline injection group (AR: ST36 acupoint, dose: not reported). However, the mice injected with the single-low-dose BVA (0.25 mg/kg) on the ST36 acupoint did not demonstrate an effect at any time point.

##### Intervertebral Disc Disease-Induced Neurological Pain

Intervertebral disc disease (IDD) is a spinal disorder that causes neurological pain and dysfunction [51]. A single-blind RCT involving 36 adult canines with IDD pain was divided into two groups. The control group received CT with oral prednisone (1 mg/kg) together with the nonsteroidal anti-inflammatory drug (NSAID) carprofen (2.2 mg/kg) and ranitidine (2 mg/kg). The experimental group was subcutaneously injected with BV (5 mg/kg) into the LI4, SI3, KI3, ST36, BL23, BL40, GB30, GB34, LR3, GV1, Baihui, and Ashi acupoints, and also received control treatment. The myelopathy scoring system was significantly improved in the moderate (*p* = 0.001) and severe groups (*p* = 0.014) but was not significantly reduced in the mild group (*p* = 0.59). The results suggest that BVA plus CT is more effective for IDD pain treatment than CT alone.

### 3.4. Experimental Studies of Post-Ischaemic Pain

In the study reported by Lee et al. [52], a chronic post-ischaemic pain model was induced in male C57/Bl6 mice with isoflurane by placing a tight-fitting O-ring at the left ankle for 3 h. The significant effect persisted for different durations in the BVA group (AR: dorsum of the ipsilateral hind paw, dose: 1.0 mg/kg) compared with the saline injection group: 90 min for the single BV injection group, 120 min for the double BV injection group, and 180 min for the triple BV injection group. The results indicate that repeated BV injections amplify the anti-allodynic effect in mice with chronic post-ischaemic pain.

### 3.5. Experimental Studies of Prostatic Pain

Complete Freund’s adjuvant (CFA)-induced prostate pain model rats were injected with melittin or saline into both the right and left lobes of the prostate, and one rat group received a sham operation [53]. Melittin is a major biological component of BV, accounting for 40%–60% of its complete dry weight [36]. Compared to the sham operation group, the melittin group (dose: 0.05 mg melittin in a volume of 0.2 mL saline) showed significant effects in the threshold of mechanical pain at 12 days (*p* < 0.01) and 18 days (*p* < 0.01). The melittin group showed a significant reduction in prostatic pain at 18 days (*p* < 0.05) compared with the saline injection group (volume: 0.2 mL). This study suggests that melittin may be used as a treatment for chronic prostatic pain.

## 4. Therapeutic Mechanisms of BV on Pain Management

The therapeutic mechanisms of BVA reported in experimental studies and clinical trials are as follows.

(1) In cases of musculoskeletal pain, seven clinical studies [14,15,16,17,18,19,20] did not report the analgesic mechanism of BVA.

(2) In cases of inflammatory pain, interleukin-6 (IL-6) and tumour necrosis factor-α (TNF-α) levels were found to be decreased by BVA (Species: *Apis dorsata* *(A. dorsata)*, AR: oral route, dose: 2.0 mg/kg) in a collagen type-II induced arthritis pain model [22]. BVA (Species: *Apis mellifera* *(A.* *mellifera*), AR: dorsal route, dose: 0.25 mg/kg in a volume of 50 µL) restores altered activity of basic (APN) in synovial fluid (SF), soluble fraction (S) from synovial tissue (ST), and dipeptidyl peptidase IV (DPPIV) in solubilised membrane-bound fraction (M) from peripheral blood mononuclear cells (PBMCs) [23]. BVA attenuates body mass (BM) and normalizes TNF-α independently of methotrexate, and BVA is a source for obtaining the DPPIV enzyme [23]. BVA (Species: *A. mellifera*, AR: ST36 acupoint, dose: 0.8 mg/kg, BV dissolved in a volume of 20 µL saline) on spinal astrocyte activation is mediated by the activation of both α-2A and α-2C adrenoceptors, which in turn results in a potent analgesic effect [24]. BVA (Species: *A. mellifera*, AR: ST36 acupoint, dose: 0.8 mg/kg (1 K, diluted by saline with ratio of 1:1000)) in an inflammatory pain model could be significantly improved by modulation of adrenal medulla-derived epinephrine, and this effect is mediated by peripheral *β*-adrenoceptors [25]. BVA (Species: *A. mellifera*, AR: ST36 acupoint, dose: 1.0 mg/kg) can relieve collagen type-II-induced pain via the partial involvement of the δ-opioid and α2-adrenergic receptors [26].

(3) In cases of neuropathic pain, BVA (Species: *A. mellifera*, AR: GB39, LI4, LV3, and SJ5 acupoints, dose: 0.1 mg/mL BVA was injected, total volume of 9.6 mL in 6 sessions) may demonstrate functions via mechanisms similar to those observed with norepinephrine reuptake inhibitors or adrenergic reuptake inhibitors [30]. Combination treatment with BVA (Species: *A. mellifera*, AR: ST36 acupoint, dose: 1.0 mg/kg, BV dissolved in a volume of 20 μL PBS) and venlafaxine (AR: oral route, dose: 40 mg/kg, venlafaxine dissolved in a saline) can help relieve paclitaxel-induced cold and mechanical allodynia through spinal 5-HT1/5-HT2, 5-HT3, and α2-adrenergic receptors [36]. The combined effect of BVA (Species: *A. mellifera*, 1.0 mg/kg was injected into ST36 acupoint in a volume of 20 μL) and morphine (AR: intraperitoneal route, dose: 2.0 mg/kg in a volume of 0.2 mL) on oxaliplatin-induced cold and mechanical allodynia is mediated by a spinal opioidergic and 5-HT3 receptors [37]. BVA (Species: *A. mellifera*, AR: ST36 acupoint, dose: 1.0 mg/kg, BV dissolved in PBS at concentrations of 1 mg/mL) may attenuate oxaliplatin-induced neuropathic pain by altering the action potential threshold in A-fibre dorsal root ganglia neurons [43]. BVA (Species: *A. mellifera*, AR: ST36 acupoint, dose: 1.0 mg/kg, BV dissolved in volume of 50 μL saline)-induced analgesia is mediated by the descending noradrenergic pathway, which mainly originates from the locus coeruleus (LC) [46].

PLA_2_ (Species: *A. mellifera*, AR: intraperitoneal route, dose: 0.2 mg/kg, PLA_2_ dissolved in a PBS) can help reduce spinal nerve injury-induced mechanical and cold allodynia through the activation of spinal α1-adrenergic receptors [33]. PLA_2_ (Species: *A. mellifera*, AR: not reported, dose: 0.2 mg/kg, PLA_2_ dissolved in a PBS) reduces oxaliplatin-induced mechanical and cold allodynia through activation of α2-adrenergic receptors [38]. PLA_2_ (Species: *A. mellifera*, AR: intraperitoneal route, dose: 0.2 mg/kg) may prevent oxaliplatin-induced mechanical and cold allodynia by suppressing immune responses in the dorsal root ganglia (DRG) by tregs [39]. 

Melittin (Species: *A. mellifera*, AR: ST36 acupoint, dose: 0.5 mg/kg, melittin dissolved in a saline) demonstrates an analgesic effect on oxaliplatin-induced peripheral neuropathy, and its effect is mediated by activation of the spinal α1 and α2-adrenergic receptors [42].

(4) In cases of mechanical allodynia, BVA (Species: *A. mellifera*, AR: ST36 acupoint, dose: 1.0 mg/kg, BV dissolved in a PBS) demonstrates potent suppressive effects in paclitaxel-induced mechanical allodynia, which is mediated by the spinal α2-adrenergic receptor [35]. 

The anti-allodynic effect of BVA (Species: *A. mellifera*, AR: ST36 acupoint, dose: 0.1 mg/kg, BV dissolved in a volume of 20 μL saline) is related to the activation of peripheral nerves in a BVA-injected site, and it is also meditated by the activation of alpha-2 adrenoceptors and opioid receptors in the spinal cord [40]. Repetitive BVA (Species: *A. mellifera*, AR: ST36 acupoint, dose: 0.1 mg/kg, BV dissolved in a volume of 20 μL saline) injected 18 times gradually and significantly decreases oxaliplatin-induced mechanical allodynia and recovers the loss of intraepidermal nerve fibres (IENFs) in neuropathic mice through an α2-adrenoceptor mechanism [41]. The analgesic effects of repetitive BVA (Species: *A. mellifera*, AR: ST36 acupoint, dose: 0.25 mg/kg, BV dissolved in a volume of 50 μL saline) injected seven times are related to the activation of the LC noradrenergic system and a decrease in spinal phosphorylation [44]. The repetitive application of BVA (Species: *A. mellifera*, AR: ST36 acupoint, dose: 0.25 mg/kg, BV dissolved in a volume of 50 μL saline) suppressed SCI-induced mechanical allodynia, which is mediated by the suppression of spinal astrocytes or microglial activation [45].

(5) In cases of cold allodynia, BVA (Species: *A. mellifera*, AR: GV3 acupoint, dose: 0.25 mg/kg, BV dissolved in a volume of 0.05 cc saline) relieves oxaliplatin-induced cold allodynia through activation of the serotonergic system, especially spinal 5-HT3 receptors [47]. Spinal α4β2 receptors mediate the suppressive effect of BVA (Species: *A. mellifera*, AR: GV3 acupoint, dose: 1.0 mg/kg, BV dissolved in a volume of 0.05 cc saline) on oxaliplatin-induced cold allodynia [48]. The anti-allodynic effect of BVA (Species: *A. mellifera*, AR: GV3 acupoint, dose: 1.0 mg/kg, BV dissolved in a volume of 0.05 cc saline) on oxaliplatin-induced cold allodynia is mediated by the noradrenergic system, and not the opioid system [49]. BVA (Species: *A. mellifera*, AR: ST36 acupoint, dose: 2.5 mg/kg, BV dissolved in a volume of 50 μL saline) could reduce cold allodynia through activation of spinal α2-adrenoceptors in chronic constrictive injury-induced rats [50].

(6) In cases of post-ischaemic pain, the anti-allodynic effect of BVA (Species: *A. mellifera*, AR: dorsum of the ipsilateral hind paw, dose: 1.0 mg/kg BV injected 1, 2, and 3 times) has been objectively demonstrated through a decrease in the increased expression of neurokinin type 1 (NK-1) receptors in the DRG [53].

(7) In cases of prostatic pain, melittin (Species: *A. mellifera*, AR: both right and left lobes of the prostate, dose: 0.05 mg melittin in a volume of 0.2 mL saline) alleviates CFA-induced prostatitis pain by suppressing cyclooxygenase-2 expression [53].

## 5. Discussion

In clinical settings, the existing literature suggests that BVA monotherapy or a combination with CT (e.g., NSAID and PT) can help improve musculoskeletal and neuropathic pain. Additionally, only minor adverse events such as pruritus, burning sensation, local swelling, and aching were observed in the BVA treatment groups, and fatal reactions such as anaphylaxis did not occur [56]. Anaphylaxis was not observed in clinical trials with relatively small sample sizes, because of a low incidence of 0.014% [57]. Patients who have a high risk of developing anaphylaxis or hypersensitivity were excluded according to the skin tests, and the history of patients was documented [35]. The safety of BVA is a crucial factor in deciding the applicability of BV as a therapeutic agent [11]. Therefore, when using BVA in humans, it is only recommended for use after determining the possibility of occurrence of allergic reactions by considering the conduction of a skin test and by documenting treatment history and family history.

The incidence of side effects of bee sting therapy (BST) was 7.5–98.83%, with a large variation [58,59,60]. In case of BVA, the incidence of AEs was 0.03–18.18% [61,62]. In order to use BV clinically, safety must be guaranteed. The BST is a treatment that stimulates the skin with live bee sting [35]. The BVA is the injection of purified and diluted BV from worker bees into the acupuncture points of human [35]. Thus, BVA corresponds to a treatment developed to produce a therapeutic effect while reducing side effects when clinically applying BV compared to BST. However, there may be deviations in the occurrence of side effects due to dilution concentration, injection dose, and sensitivity to BV. Further studies on prediction factors to prevent AEs of BVA will be investigated.

In most clinical studies, it was reported that BVA was diluted with saline at a certain ratio and injected to patients. In experimental studies, BVA was used diluted in saline or PBS, or used without dilution. It is possible that undiluted BVA was injected into mice or rats, or diluted BVA was injected but not mentioned in the article. Therefore, it is necessary to specify the dilution method and used concentration of BVA in future clinical or experimental studies. In addition, we look forward to future studies on any differences in effectiveness and safety depending on the concentration of BVA and the substances used for dilution.

Among the 40 studies included in this review, 39 were conducted using BV, PLA_2_, and melittin extracted from *A. mellifera*. Honeybees (*A. mellifera*) can be found worldwide, and there is considerable genetic diversity in global *A. mellifera* populations [63,64]. Additionally, depending on the environmental differences such as the region, climate, and flower distribution within the same country, differences in the composition and concentration of BV may be observed. As BV is a natural toxin, a certain level of standardisation is necessary for its use as a raw material for drug development and clinical and experimental studies.

The pain reduction effects and mechanisms of collagen-, adjuvant-, and formalin-induced inflammatory pain in a mouse model [22,23,24,25,26] were confirmed through animal studies. However, all included studies confirmed the treatment effect of inflammatory pain following a single BVA treatment. In future studies, the number of administrations and treatment periods should be extended to identify conditions that can help produce the optimal treatment effect. In another study [24], high-concentration BVA (0.8 mg/kg) exerted a significant analgesic effect compared to low-concentration BVA (0.08 mg/kg), and in another study [26], low-concentration BVA (1 mg/kg) exerted a significant analgesic effect. It has been reported to be more effective than BVA (2 mg/kg). The concentration of venom correlates to both therapeutic efficacy and adverse allergic and toxic reactions. We anticipate the conduction of further studies for examination of the effect of BV on the treatment of inflammatory pain, considering BV concentration.

Prior to 2010, pain-related clinical studies of BVA focused on osteoarthritis, rheumatoid arthritis, and musculoskeletal pain (e.g., low back, neck, and shoulder pain, acute ankle or wrist sprain, and pain of a herniated lumbar disc) [13]. In animal studies, most experimental studies have been conducted using inflammatory nociception model mice induced by collagen, adjuvant, and formalin, and only one study was conducted using neuropathic pain model mice induced by chronic constriction injury [13]. On the other hand, in the last 10 years, clinical studies have been conducted on various aspects and types of neuralgia, and experimental studies have been conducted on mechanical and cold allodynia. Studies have been conducted on the following: (1) post-stroke pain [27], chemotherapy-induced peripheral neuropathic pain [29,30], complex regional pain syndrome [31], refractory postherpetic neuralgia [32], and intervertebral disc disease-induced neurological pain in clinical fields [51]; (2) mechanical allodynia induced by SNL [33,34], CCI [44,45,46], and chemotherapy drugs including oxaliplatin [37,38,39,40,41,42,43] and paclitaxel [35,36] in animal studies; (3) cold allodynia induced by SNL [34,35], CCI [50], and chemotherapy drugs including oxaliplatin [37,38,39,42,43,47,48,49], paclitaxel [36], and vincristine [46] in animal experimental studies. Multiple studies have been conducted on neuropathic pain induced by chemotherapy drugs. Approximately 66% of the cancer patients experience mild to severe pain during treatment [65,66]. This suggests that BVA could be utilized to treat chemotherapy-induced pain, and we anticipate the conduction of further research on various chemotherapy-induced pain conditions. Pain is one of the most common symptoms in humans, and pain, especially neuropathic pain and chemotherapy-induced pain, can affect daily life and economic activities [67]. It is not necessary to utilize only one method for the treatment of pain, nor is it necessary to change the conventional drugs to BVA. As BVA is not consumed orally, and is instead injected into acupoints with a syringe, it can be considered as a viable alternative intervention for patients presenting with pain who encounter problems with oral administration. The treatment effects and mechanisms of anti-nociception have been well characterised in articles published on the effects of BVA on pain. We look forward to using BVA as one of the treatments that can be used to treat pain; additionally, we aim to investigate various pain conditions in clinical, experimental analyses using BVA.

BV injection on acupoint ST36 has been reported in ten experimental studies [35,36,37,40,41,42,43,45,46,49]. The ST36 is located on the leg, 3 cm below the knee, and its functions in Oriental medicine involve the fortification of the stomach and spleen, the replenishment of qi and nourishing blood, clearance and activation of meridians, and nourishment of the channels [68,69]. Although the target disease, treatment concentration, and dose of BVA are extremely important, the treatment effect may vary depending on the acupuncture point at which the treatment is performed from the perspective of Oriental medicine. Lim et al. [49] assessed the analgesic effects of oxaliplatin-induced cold allodynia by administering BV at three acupoints (GV3, LI11, and ST36), and found that the duration of treatment effect was statistically significant in the GV3 group compared to other groups. As BVA is a therapeutic strategy intended for injecting BV on acupuncture points, additional research considering this point is warranted. 

## 6. Conclusions

In the past decade, numerous experimental and clinical studies have been reporting the therapeutic potential of BVA for musculoskeletal pain, inflammatory pain, neuropathic pain, post-ischaemic pain, and prostatic pain. This paper will be the basis for promoting research that will make BVA more general and safer. However, several experimental results have still not been investigated in humans. The effectiveness and safety of BVA for various types of pain have to be supported by clinical trials. In addition, in most cases, the optimal dose and frequency duration of BVA still need to be determined for optimum effectiveness and safety. 

## Figures and Tables

**Table 1:** Clinical on therapeutic application of bee venom for musculoskeletal pain.

Disease	Study Design (Number of Patient or Animal Treated by BV)	Venom/Compound/(Bee Species)	Intervention (Acupoints, Dilution Ratio, Amount of Bee Venom Use)	Main Results	Mechanism/Molecular Response	Adverse Events of BVA	Reference
Shoulder pain	Human, Systematic review (*n* = 173)	BV (*A. mellifera*)	BVA - acupoints: GB21, LI11, LI15, LI16, SI3, SI9, SI10, SI11, TE14, and EX-UE12 - concentration: 0.03–0.5 mg/mL (BV dissolved in a saline) - 1 session: 0.1–1.5 mL - total 6–16 sessions: 0.6–14.8 mL Saline injection - same acupoints, treatment sessions and dose of BVA group	BVA + CT versus saline injection + CT - significant effect of VAS (*p* = 0.03) - Significant effect of PRS (*p* = 0.009)	Not reported	Pain 2, pruritus 8, burning sensation 3 Pruritus/local swelling/redness 30, mild, generalized swelling/aching 1	[14]
Myofascial shoulder pain	Human, RCT (*n* = 50)	BV (*A. mellifera*)	BVA - acupoint: GB21 - concentration: not reported - 1 session: 1 mL - total 2 sessions: 2 mL Needle free BVA - acupoint: GB21 - concentration: not reported - 1 session: 1 mL - total 2 sessions: 2 mL	BVA versus needle free BVA - significant effect of VAS in both groups (*p* values were not reported)	Not reported	(A) BVA - fatigue 1, purpura 1, oedema 2, headache 1, cold 1, itching 16, redness 14 (B) Needle free BVA - inflammation 1, purpura 2, headache 2, redness 1	[15]
Adhesive capsulitis pain	Human, RCT (BVA(a), *n* = 22/BVA(b), *n* = 23)	BV (*A. mellifera*)	BVA group1 - acupoints: GB21, LI15, LI16, SI11, and TE14 - concentration: 0.1 mg/mL (BV dissolved in a saline) - 1 session: 0.4 mL (first visit), 0.6 mL (second visit), 0.8 mL (third visit), 1 mL (4–16 visit) - total 16 sessions: 14.8 mL BVA group2 - acupoints: GB21, LI15, LI16, SI11, and TE14 - concentration: 0.03 mg/mL (BV dissolved in a saline) - 1 session: 0.4 mL (first visit), 0.6 mL (second visit), 0.8 mL (third visit), 1 mL (4–16 visit) - total 16 sessions: 14.8 mL Saline injection - same acupoints, treatment sessions and dose of BVA group	BVA group1 + PT versus saline injection + PT - significant effect of SPADI at 8 day (*p* = 0.025) and 12 day (*p* = 0.014) - significant effect of VAS at rest score at 8th week (*p* = 0.048), during motion score at 12th week (*p* = 0.029) BVA group1 + PT versus BVA group2 + PT - NS between groups	Not reported	BVA(a) and BVA(b) - slight pruritus, local swelling, and/or redness 30, BVA(a) - mild, generalized swelling and aching 1	[16]
Adhesive capsulitis pain	Human, One year follow-up study of previous RCT [16], (BVA(a), *n* = 20/ BVA(b), *n* = 22)	BV (*A. mellifera*)	BVA group1 - acupoints: GB21, LI15, LI16, SI11, and TE14 - concentration: 0.1 mg/mL (BV dissolved in a saline) - 1 session: 0.4 mL (first visit), 0.6 mL (second visit), 0.8 mL (third visit), 1 mL (4–16 visit) - total 16 sessions: 14.8 mL BVA group2 - acupoints: GB21, LI15, LI16, SI11, and TE14 - concentration: 0.03 mg/mL (BV dissolved in a saline) - 1 session: 0.4 mL (first visit), 0.6 mL (second visit), 0.8 mL (third visit), 1 mL (4–16 visit) - total 16 sessions: 14.8 mL Saline injection - same acupoints, treatment sessions and dose of BVA group	BVA group1 + PT versus saline injection + PT - significant effect of SPADI at 1 year (*p* = 0.043) BVA group1 + PT versus BVA group2 + PT versus saline injection + PT - NS of pain VRS among 3 groups	Not reported	Not reported	[17]
Chronic low back pain	Human, RCT (*n* = 27)	BV (*A. mellifera*)	BVA - acupoints: BL23, BL24, BL25, GB30, GV3, GV4, and GV5 - concentration: 0.05 mg/mL (BV dissolved in a saline) - 1 session: 2 mL (first week), 4 mL - (second week), 8 mL (third week) - total 6 sessions: 28 mL Saline injection - same acupoints, treatment sessions and dose of BVA group	BVA plus NSAID versus saline injection plus NSAID - significant effect of VAS for pain intensity (*p* = 0.0486)	Not reported	Itching/sensation 4, headache 1, generalized myalgia 1	[18]
Chronic low back SOFJApain	Human, RCT (*n* = 60)	BV (*A. mellifera*)	BVA - acupoints: BL23, BL24, and BL25 - concentration: 0.05 mg/mL (BV dissolved in a saline) - 1 session: 0.6 mL - total 8 sessions: 4.8 mL Saline injection - same acupoints, treatment sessions and dose of BVA group	BVA versus saline injection - significant effect of VAS for pain intensity (*p* = 0.0087)	Not reported	pruritus 15, skin flare 5, oedema 4, skin rash 2	[19]
Low back pain	Human, Retrospective study (*n* = 524)	BV (*A. mellifera*)	BVA - acupoint: 4–5 acupoints around the lumbar spine - concentration: 0.1 mg/mL (BV dissolved in a saline) - 1 session: 0.5–1 mL - total treatment sessions: 2.3 ± 1.8	BVA + herbal medicine + acupuncture + chuna - average reduction in NRS was 3.18–2.29 (95% confidence interval [CI], 2.99–3.38)	Not reported	Allergic reactions 8	[20]

Abbreviations: *A. mellifera: Apis* *mellifera*, BV: bee venom, BVA: bee venom acupuncture, CT: conventional treatment, NRS: numeral rating scale, NS: no significant effects, NSAID: non-steroidal anti-inflammatory drugs, PT: physiotherapy, RCT: randomized controlled trials, SPADI: shoulder pain and disability index, VAS: visual analogue scale, VRS: verbal rating scale.

**Table 2:** Clinical studies on therapeutic application of bee venom for inflammatory pain.

Disease	Study Design (Number of Patient or Animal Treated by BV)	Venom/Compound/(Bee Species)	Intervention (Dilution Ratio, Amount of Bee Venom Use)	Main Results	Mechanism/Molecular Response	Adverse Events of BVA	Reference
Knee osteoarthritis pain	Human, RCT (*n* = 361)	BV (*A. mellifera*)	BVA - acupoints: BL40, BL19, BL 21, BL 23, BL 25, BL27, ST34, 5 on each knee (knee top), eye-1 medial, and eye-2 lateral - concentration: bee venom powder 1 mg and 1 mL 0.5% lidocaine were mixed - 1 session: 1.2 mL (1–3 weeks), 1.5 mL (4–12) - total 12 sessions: 17.1 mL histamine injection - concentration: 0.275 mg/mL - same acupoints, treatment sessions and dose of BVA group	BVA versus histamine injection - significant effect of WOMAC pain score (*p* = 0.0010)	Not reported	AEs 177, injection site AEs 15	[21]

Abbreviations: *A. mellifera: Apis* *mellifera*, AE: adverse event, BV: bee venom, BVA: bee venom acupuncture, Ontario and McMaster Universities Osteoarthritis index (WOMAC), RCT: randomized controlled trials.

**Table 3:** Experimental studies on therapeutic application of bee venom for inflammatory pain.

Disease	Study Design (Number of Patient or Animal Treated by BV)	Venom/Compound/(Bee Species)	Intervention (Dilution Ratio, Amount of Bee Venom Use)	Main Results	Mechanism/Molecular Response	Adverse Events of BVA	Reference
Arthritis pain	Male Wistar rats (190–210 g), FCA and collagen type II induced arthritis model (E1, *n* = 6, E2, *n* = 6)	BV (*A. dorsata*)	- E1: FCA-induced rats with BV injected once (AR: right hind paw, dose: 1 mg/200 g) - E2: collagen type II-induced rats with BV injected once (AR: right hind paw, dose: 1.0 mg/200 g) - AC1: FCA-induced rats with Indomethacin administered once (AR: oral route, dose: 2.0 mg/kg) - AC2: collagen type II-induced rats with Indomethacin administered once (AR: oral route, dose: 2.0 mg/kg) - NC1: FCA-induced rats with saline injected once (AR: right hind paw, dose: 0.1 mL) - NC2: collagen type II-induced rats with saline injected once (AR: right hind paw, dose: 0.1 mL)	Arthritis pain (dorsal flexion pain score) - (E1 vs. NC1) NS (after 7 day), NS (after 14 day), *p* < 0.05 (after 21 day), and *p* < 0.01 (after 28 day) - (AC1 vs. NC1) NS (after 7 day), NS (after 14 day), *p* < 0.05 (after 21 day), and *p* < 0.01 (after 28 day) - (E2 vs. NC2) NS (after 9 day), NS (after 18 day), *p* < 0.05 (after 27 day), and *p* < 0.05 (after 36 day) - (AC2 vs. NC2) NS (after 9 day), NS (after 18 day), *p* < 0.05 (after 27 day), and *p* < 0.05 (after 36 day)	- BV reduces proinflammatory markers such as TNF-a and IL-6	Not reported	[22]
Arthritis pain	Male Wistar rats (150–160 g), collagen type II induced arthritis pain (E1, *n* = 5/E2, *n* = 3)	BV (*A. mellifera*)	- E1: collagen type II induced rats with BV subcutaneously injected once (AR: dorsal route, dose: 0.25 mg/kg in a volume of 50 µL) - E2: E1 group + methotrexate subcutaneously injected once (AR: dorsal route, dose: 0.3 mg/kg in a volume of 300 µL) - AC1: collagen type II induced rats with saline subcutaneously injected once (AR: dorsal route, volume: 50 µL) - AC2: collagen type II-induced rats with methotrexate subcutaneously injected once (AR: dorsal route, dose: 0.3 mg/kg in a volume of 300 µL) - NC: Healthy rats with saline subcutaneously injected once (AR: dorsal route, volume: 50 µL)	Arthritis pain (mechanical threshold of hyperalgesia) - (AC1 vs. NC) decrease in AC1 compared with NC - (E1 vs. NC) NS between E1 and NC - (AC2 vs. NC) decrease in AC2 compared with NC - (E2 vs. NC) decrease in E2 compared with NC	- BV restores APN in SF and in soluble fraction from ST, and DPPIV in solubilized membrane-bound fraction from PBMCs	Not reported	[23]
Inflammatory pain	Male ICR mice (20–25 g), formalin induced inflammatory pain (E1, *n* = 8/E2, *n* = 8)	BV (*A. mellifera*)	- E1: formalin-induced mice with BV subcutaneously injected once before formalin injection (AR: ST36 acupoint, dose: 0.8 mg/kg, BV dissolved in a volume of 20 µL saline) - E2: formalin-induced mice with BV subcutaneously injected once before formalin injection (AR: ST36 acupoint, dose: 0.08 mg/kg, BV dissolved in a volume of 20 µL saline) - NC: formalin induced mice with saline subcutaneously injected once before formalin injection (AR: ST36 acupoint, volume: 20 µL)	Inflammatory pain (paw licking time) - (E1 vs. NC) NS (first 10 min), and *p* < 0.001 (subsequent 20 min) - (E2 vs. NC) NS (first 10 min), and NS (subsequent 20 min)	- BVA produces a potent anti-nociceptive effect via the activation of spinal α-2 adrenoceptors	Not reported	[24]
Inflammatory pain	Male ICR mice (25–30 g), formalin induced inflammatory pain (E, *n* = 8/AC1, *n* = 8/AC2, *n* = 8)	BV (*A. mellifera*)	- E: formalin-induced mice with BV (AR: ST36 acupoint, dose: 0.8 mg/kg (1 K, diluted by saline with ratio of 1:1000)) and saline (AR: ST36 acupoint, volume: 100 µL) subcutaneously injected once after formalin injection - AC1: formalin-induced mice with BV (AR: ST36 acupoint, dose: 0.8 mg/kg (1 K, diluted by saline with ratio of 1:1000) and hydroxydopamine (AR: intraperitoneal route, volume: 100 µL) injected once after formalin injection - AC2: formalin-induced mice with BV (AR: ST36 acupoint, dose: 0.8 mg/kg (1 K, diluted by saline with ratio of 1:1000) and epinephrine (AR: intraperitoneal route, volume: 100 µL) injected once after formalin injection - NC: formalin- induced mice with saline subcutaneously injected once after formalin injection (AR: ST36 acupoint, volume: 100 µL)	Inflammatory pain (paw licking time) - (E vs. NC) NS (first 10 min), and *p* < 0.001 (subsequent 20 min) - (AC1 vs. NC) NS (first 10 min), and *p* < 0.001 (subsequent 20 min) - (E vs. AC1) NS (first 10 min), and NS (subsequent 20 min) - (E vs. NC) NS (first 10 min), and *p* < 0.001 (subsequent 20 min) - (AC2 vs. NC) NS (first 10 min), and NS (subsequent 20 min) - (E vs. AC2) NS (first 10 min), and *p* < 0.01 (subsequent 20 min)	- BVA performed in combination with administration of peripheral β-adrenoceptor antagonists would be a potential novel strategy for the pain management	Not reported	[25]
Osteoarthritis pain	Male Sprague-Dawley rats (200–250 g), collagen type II induced osteoarthritis pain (E1, *n* = 10/E2, *n* = 10/ AC, *n* = 10)	BV (*A. mellifera*)	- E1: collagen type II-induced rats with BV subcutaneously injected once (AR: ST36 acupoint, dose: 1.0 mg/kg) - E2: collagen type II-induced rats with BV subcutaneously injected once (AR: ST36 acupoint, dose: 2.0 mg/kg) - AC: collagen type II induced rats with BV injected once (AR: intraperitoneal route, dose: 1.0 mg/kg)	Osteoarthritis pain (tail flick latency) - (E1 vs. AC) *p* < 0.05 (after 10 min), *p* < 0.01 (after 20 min), *p* < 0.001 (after 30 and 45 min), and *p* < 0.01 (after 60 min) - (E1 vs. E2) *p* < 0.05 (after 10 min), *p* < 0.05 (after 20 min), *p* < 0.01 (after 30 min), *p* < 0.01 (after 45 min), and *p* < 0.01 (after 60 min)	- BVA reduced pain through the partial involvement of the δ-opioid and α2-adrenergic receptors	Not reported	[26]

Abbreviations: *A*. *dorsata: Apis dorsata*, *A. mellifera: Apis* *mellifera*, AC: active control group, APN: altered activity of basic, AR: administration route, BV: bee venom, BVA: bee venom acupuncture, DPPIV: dipeptidyl peptidase IV, E: experimental group, FCA: Freund’s complete adjuvant, IL-6: interleukin-6, NC: normal control group, NS: no significant effects, PBMCs: peripheral blood mononuclear cells, SF: synovial fluid, ST: synovial tissue, TNF-α: tumour necrosis factor- α.

**Table 4:** Clinical studies on therapeutic application of bee venom for neuropathic pain.

Disease	Study Design (Number of Patient or Animal Treated by BV)	Venom/Compound/(Bee Species)	Intervention (Dilution Ratio, Amount of Bee Venom Use)	Main Results	Mechanism/Molecular Response	Adverse Events of BVA	Reference
Post-stroke shoulder pain	Human, systematic review (*n* = 75)	BV (*A. mellifera*)	BVA - acupoints: EX-UE70, GB21, LI11, LI15, SI3, SI9, SI10, SI11, and TE14 - concentration: 0.1–0.5 mg/mL (BV dissolved in a saline) - 1 session: 0.1–1.5 mL - total 6–12 sessions: 0.9–13.5 mL Saline injection - same acupoints, treatment sessions and dose of BVA group	BVA versus saline injection - significant effect of VAS (*p* = 0.02)	Not reported	Pain 2, pruritus 8, burning sensation 3	[27]
Central post-stroke pain	Human, RCT (*n* = 8)	BV (*A. mellifera*)	BVA - acupoints: GB21, GB31, GB39, LI11, LI15, and ST36 - concentration: not reported - 1 session: 0.3 mL - total 6 sessions: 1.8 mL Saline injection - same acupoints, treatment sessions and dose of BVA group	BVA versus saline injection - significant effect of VAS (*p* = 0.009)	Not reported	No adverse events	[28]
CIPN	Human, Case series (*n* = 4)	BV (*A. mellifera*)	BVA - acupoints: Ba Xie and Ba Feng (located on the extremities of the hands and feet proximal to the margins of the webs between all 5 fingers and toes) - concentration: 0.1 mg/mL (BV dissolved in a saline) - 1 session: 1.6 mL - total 3 sessions: 4.8 mL	Improved of VAS (8.75 to 2.75)	Not reported	No adverse events	[29]
CIPN	Human, Case studies (*n* = 11)	BV (*A. mellifera*)	BVA - acupoints: GB39, LI4, LV3, and SJ5 - concentration: 0.1 mg/mL (BV dissolved in a saline) - 1 session: 1.6 mL - total 6 sessions: 9.6 mL	Significant effect of VAS at after 6 times (*p* < 0.05) and 3 weeks after final treatment (*p* < 0.01)	BVA may work in the similar mechanisms as the norepinephrine reuptake inhibitors or adrenergic reuptake inhibitors	Swelling and itchiness 2, mild fever 1	[30]
Complex regional pain syndrome	Human, Case report (*n* = 1)	BV (*A. mellifera*)	BVA - acupoint: GB43 - concentration: not reported - 1 session: 0.15 mL (first visit), 0.2 mL (second visit), 0.3 mL (3–8 visit), 0.4 mL (9–14 visit) - total 14 sessions: 4.55 mL	- Worst level of NRS was reduced from 8 to 0 - Usual level of NRS was reduced from 5 to 0 - Best level of NRS was reduced from 3 to 0	Not reported	No adverse events	[31]
Refractory postherpetic neuralgia	Human, Case report (*n* = 1)	BV (*A. mellifera*)	BVA - acupoint: not reported - concentration: 0.03 mg/mL (BV dissolved in a saline) - 1 session: 0.1 mL for each site total volume did not exceed 1 mL - total 4 sessions: total volume did not exceed 4 mL	NRS was reduced from 8 to 2	Not reported	Itchiness	[32]

Abbreviations: *A. mellifera: Apis* *mellifera*, BV: bee venom, BVA: bee venom acupuncture, CIPN: chemotherapy-induced peripheral neuropathic pain, NRS: numeral rating scale, RCT: randomized controlled trials, VAS: visual analogue scale.

**Table 5:** Experimental studies on therapeutic application of bee venom for neuropathic pain.

Disease	Study Design (Number of Patient or Animal Treated by BV)	Venom/Compound/(Bee Species)	Intervention (Dilution Ratio, Amount of Bee Venom Use)	Main Results	Mechanism/Molecular Response	Adverse Events of BVA	Reference
Mechanical allodynia	8-week male Sprague-Dawley rats, SNL injured mechanical allodynia (*n* = 10)	PLA_2_ (*A. mellifera*)	- E: SNL-injured rats with PLA_2_ injected once (AR: intraperitoneal route, dose: 0.2 mg/kg, PLA_2_ dissolved in a PBS) - NC: SNL-injured rats with PBS injected once (AR: intraperitoneal route, dose: 0.2 mg/kg)	Mechanical allodynia (von Frey test) - (E vs. NC) NS (after 1 day), and *p* < 0.001 (after 2 day)	- α1-adrenergic receptors activation could inhibit the mechanical allodynia induced by nerve injury	Not reported	[33]
Mechanical and cold allodynia	Male Sprague Dawley rats (180–200 g), SNL injured mechanical and cold allodynia (E1, *n* = 7/E2, *n* = 7)	BV (*A. mellifera*)	- E1: SNL-injured rats with BV injected once (AR: peri-neural sheath of the L5 and L6 spinal nerves, dose: 0.1 mg/kg, BV dissolved in 0.9% saline at concentrations of 2 μg/μL) - E2: SNL-injured rats with BV injected once (AR: peri-neural sheath of the L5 and L6 spinal nerves, dose: 0.05 mg/kg, BV dissolved in 0.9% saline at concentrations of 1 μg/μL) - NC: SNL-injured rats with saline injected once (AR: peri-neural sheath of the L5 and L6 spinal nerves, dose: not reported)	Mechanical allodynia (von Frey test) - (E1 vs. NC) *p* < 0.05 (after 3, 5, 7, 9, 13 day) - (E2 vs. NC) *p* < 0.05 (after 3, 5, 7, 9, 13, 21, 29 days), Cold allodynia (acetone test) - (E1 vs. NC) *p* < 0.05 (after 3, 5, 7, 9, 13, 17, 21, 25, 29 days) - (E2 vs. NC) *p* < 0.05 (after 3, 5, 7, 9, 13, 17, 21, 25, 29 days) Cold allodynia (cold plate test) - (E1 vs. E2) *p* < 0.05 (after 7, 9, 13, 17, 21, 25 days)	(mechanical allodynia) - not reported. (cold allodynia) - The expression of TRPM8 receptors was significantly reduced after BVA pretreatment, indicating that both TRPA1 and TRPM8 are involved in the development of cold allodynia in the SNL injury model	Not reported	[34]
Mechanical allodynia	6-week male Sprague-Dawley rats (180–210 g), paclitaxel induced mechanical allodynia (*n* = 14)	BV (*A. mellifera*)	- E: paclitaxel-induced rats with BV injected once (AR: ST36 acupoint, dose: 1.0 mg/kg, BV dissolved in a PBS) - NC: paclitaxel-induced rats with PBS injected once (AR: ST36 acupoint, dose: not reported)	Mechanical allodynia (von Frey test) - (E1 vs. NC) *p* < 0.05 (after 1 h), *p* < 0.01 (after 2 h), NS (after 4 h), and NS (after 6 days)	- The action of spinal α2-adrenergic receptor, but not α1-adrenergic receptor, is involved in the mechanism of analgesic effect.	Not reported	[35]
Mechanical and cold allodynia	Male C57BL/6j mice (18–20 g), paclitaxel induced mechanical and cold allodynia (E1, *n* = 16/E2, *n* = 16)	BV (*A. mellifera*)	- E1: paclitaxel-induced mice with BV (AR: ST36 acupoint, dose: 1.0 mg/kg, BV dissolved in a volume of 20 μL PBS) and saline (AR: ST36 acupoint, dose: same dose of BV) subcutaneously injected once - E2: paclitaxel-induced mice with BV (AR: ST36 acupoint, dose: 1.0 mg/kg, BV dissolved in a volume of 20 μL PBS) and venlafaxine (AR: oral route, dose: 40 mg/kg, venlafaxine dissolved in a saline) administered once - AC: paclitaxel-induced mice with venlafaxine (AR: oral route, dose: 40 mg/kg, venlafaxine dissolved in a saline) and PBS (AR: ST36 acupoint, dose: not reported) administered once - NC: paclitaxel-induced mice with PBS (AR: ST36 acupoint, dose: not reported) and saline (AR: ST36 acupoint, dose: not reported) injected once	Mechanical allodynia (von Frey test) - (E1 vs. NC) *p* < 0.05 (after 60 min), and *p* < 0.01 (after 120 min) - (E2 vs. NC) *p* < 0.001 (after 60 min), *p* < 0.01 (after 120 min), and *p* < 0.05 (after 180 min) - (AC vs. NC) *p* < 0.01 (after 60 min) Cold allodynia (acetone test) - (E1 vs. NC) *p* < 0.001 (after 60 min), and *p* < 0.05 (after 120 min) - (E2 vs. NC) *p* < 0.001 (after 60, 120, 180 min) - (AC vs. NC) *p* < 0.001 (after 60 min), and *p* < 0.01 (after 120 min) - (E2 vs. E1) *p* < 0.01 (after 60, 120, 180 min)	(mechanical and cold allodynia) - Co-treatment with BVA and venlafaxine provided longer-lasting and additive analgesic action via spinal α2-adrenergic, 5-HT1/5-HT2, and 5-HT3 receptors	Not reported	[36]
Mechanical and cold allodynia	6–8-week male C57BL/6 mice, oxaliplatin induced mechanical and mechanical allodynia (*n* = 16)	BV (*A. mellifera*)	- E: oxaliplatin-induced mice with BV injected once (1.0 mg/kg was injected into ST36 acupoint in a volume of 20 μL) - AC: oxaliplatin-induced mice with morphine hydrochloride injected once (AR: intraperitoneal route, dose: 2.0 mg/kg in a volume of 0.2 mL) - NC1: oxaliplatin-induced mice with saline injected once (AR: ST36 acupoint, volume: 0.2 mL) - NC2: oxaliplatin induced mice with saline injected once (AR: intraperitoneal route, volume: 0.2 mL)	Mechanical allodynia (von Frey test) - (E+NC1 vs. NC1+NC2) *p* < 0.001 (after 30 min), and *p* < 0.01 (after 60 min) - (AC+NC2 vs. NC1+NC2) *p* < 0.001 (after 30, 60 min) - (E+AC vs. NC1+NC2) *p* < 0.001 (after 30, 60, 90 min) Cold allodynia (acetone test) - (E+NC1 vs. NC1+NC2) *p* < 0.001 (after 30 min), and *p* < 0.01 (after 60 min) - (AC+NC2 vs. NC1+NC2) NS (after 30, 60, 90 min) - (E+AC vs. NC1+NC2) *p* < 0.01 (after 30 min), and *p* < 0.001 (after 60, 90 min)	(mechanical and cold allodynia) The opioidergic and 5-HT3 receptors, but not α2-adrenergic receptors, mediate the analgesic effect at the spinal level.	Not reported	[37]
Mechanical and cold allodynia	6–8-week male C57BL/6 mice, oxaliplatin induced mechanical and cold allodynia (BV, *n* = 6/PLA2, *n* = 6)	BV (*A. mellifera*), PLA_2_ (*A. mellifera*)	- E1: oxaliplatin-induced mice with BV injected once daily for five days (AR: not reported, dose: 1.0 mg/kg, BV dissolved in a PBS) - E2: oxaliplatin-induced mice with PLA_2_ injected once daily for five days (AR: not reported, dose: 0.2 mg/kg, PLA_2_ dissolved in a PBS) - NC: oxaliplatin-induced mice with PBS injected once daily for five days (AR: intraperitoneal route, dose: not reported)	Mechanical allodynia (von Frey test) - (E1 vs. NC) *p* < 0.01 (after 2, 3 days), and *p* < 0.001 (after 4 days) - (E2 vs. NC) *p* < 0.001 (after 2, 3, 4 days), and *p* < 0.01 (after 5 days) Cold allodynia (acetone test) - (E1 vs. NC) *p* < 0.01 (after 3, 4, 5 days) - (E2 vs. NC) *p* < 0.05 (after 1, 2 day), and P < 0.001 (after 3, 4, 5, 7 days)	(mechanical and cold allodynia) - PLA_2_ treatment alleviates cold and mechanical allodynia via activation of α2-adrenergic receptors	No adverse events	[38]
Mechanical and cold allodynia	6–8-week male C57BL/6 mice, oxaliplatin induced mechanical and cold allodynia (*n* = 13)	PLA_2_ (*A. mellifera*)	- E: oxaliplatin-induced mice with PLA_2_ injected once daily for five days before oxaliplatin was administered (AR: intraperitoneal route, dose: 0.2 mg/kg) - NC1: oxaliplatin-induced mice with PBS injected once daily for five days before oxaliplatin was administered (AR: intraperitoneal route, dose: same dose of E group) - NC2: mice injected with PBS (AR: intraperitoneal route, dose: same dose of E group) once daily for five days before 5% glucose (AR: intraperitoneal route, volume: not exceed 0.5 mL) was administered	Mechanical allodynia (von Frey test) - (E vs. NC1) *p* < 0.001 (after 3 days) - (E vs. NC2) NS (after 3, 5, 7 days) Cold allodynia (acetone test) - (E vs. NC1) *p* < 0.001 (after 3, 5 days) - (E vs. NC2) NS (after 3, 5, 7 days)	(mechanical and cold allodynia) - PLA_2_ may prevent oxaliplatin-induced neuropathic pain by suppressing immune responses in the DRG by Tregs	Not reported	[39]
Mechanical allodynia	Male C57BL/6 mice (25–30 g), oxaliplatin induced mechanical allodynia (E, *n* = 6/AC, *n* = 5)	BV (*A. mellifera*)	- E: oxaliplatin-induced mice with BV injected once (AR: ST36 acupoint, dose: 0.1 mg/kg, BV dissolved in a volume of 20 μL saline) - AC: oxaliplatin-induced mice with BV injected once (AR: back point, dose: 0.1 mg/kg, BV dissolved in a volume of 20 μL saline) - NC: oxaliplatin-induced mice with saline injected once (AR: ST36 acupoint, dose: not reported)	Mechanical allodynia in right (von Frey test) - (E vs. NC) *p* < 0.001 (after 2 h) - (AC vs. NC) NS (after 1, 2, 3, 4 h) Mechanical allodynia in left (von frey test) - (E vs. NC) NS (after 1, 2, 3, 4 h) - (AC vs. NC) NS (after 1, 2, 3, 4 h)	- Anti-allodynic effect is dependent on the peripheral nerve activation in BV injected site and spinal cord α-2 adrenoceptors	Not reported	[40]
Mechanical allodynia	Male C57BL/6 mice (25–30 g), oxaliplatin induced mechanical allodynia (E, *n* = 6, AC, *n* = 5)	BV (*A. mellifera*)	- E: oxaliplatin-induced mice with BV injected once a day for 18 days (AR: ST36 acupoint, dose: 0.1 mg/kg, BV dissolved in a volume of 20 μL saline) - AC: oxaliplatin-induced mice injected once a day for 18 days (AR: back point, dose: 0.1 mg/kg, BV dissolved in a volume of 20 μL saline) - NC: oxaliplatin-induced mice with saline injected once a day for 18 days (AR: ST36 acupoint, dose: not reported)	Mechanical allodynia (paw withdrawal threshold for 1 h after BVA) - (E vs. NC) *p* < 0.01 (after 5 day), and *p* < 0.001 (after 10, 15, 20 days) - (AC vs. NC) NS (after 5, 10, 15, 20 days) Mechanical allodynia (paw withdrawal threshold prior to daily BVA) - (E vs. NC) *p* < 0.05 (after 10 days), and *p* < 0.001 (after 15, 20, 25, 30 days) - (AC vs. NC) NS (after 5, 10, 15, 20, 25, 30 days)	- Antinociceptive and restorative effects of BV injections are mediated by the activation of α-2 adrenoceptors - Oxaliplatin-induced loss of IENFs can be significantly restored by long-term treatment with BVA	Not reported	[41]
Mechanical and cold allodynia	Sprague-Dawley rats (180–200 g), oxaliplatin induced mechanical and cold allodynia (*n* = 18)	Melittin (*A. mellifera*)	- E: oxaliplatin-induced rats with melittin subcutaneously injected once (AR: ST36 acupoint, dose: 0.5 mg/kg, melittin dissolved in a saline) - NC: oxaliplatin induced rats with saline subcutaneously injected into once (AR: ST36 acupoint, dose: not reported)	Mechanical allodynia (von Frey test) - (pre and post treatment of E) *p* < 0.05 - (pre and post treatment of NC) NS Cold allodynia (acetone test) - (pre and post treatment of E) *p* < 0.01 - (pre and post treatment of NC) NS	(mechanical and cold allodynia) - The analgesic effect of melittin is mediated by activating the spinal α1 and α2-adrenergic receptor	Not reported	[42]
Mechanical and cold allodynia	Male Sprague-Dawley rats, oxaliplatin induced mechanical and cold allodynia (E1, *n* = 10/E2, *n* = 10)	BV (*A. mellifera*)	- E1: oxaliplatin-induced rats with BV subcutaneously injected once (AR: ST36 acupoint, dose: 0.1 mg/kg, BV dissolved in PBS at concentrations of 1 mg/mL) - E2: oxaliplatin-induced rats with BV subcutaneously injected once (AR: ST36 acupoint, dose: 1.0 mg/kg, BV dissolved in PBS at concentrations of 1 mg/mL) - NC: oxaliplatin-induced rats with PBS subcutaneously injected once (AR: ST36 acupoint, dose: not reported)	Mechanical allodynia (von Frey test) - (pre and post treatment of E1) *p* < 0.01 - (pre and post treatment of E2) *p* < 0.001 - (pre and post treatment of NC) NS Cold allodynia (acetone test) - (pre and post treatment of E1) NS - (pre and post treatment of E2) *p* < 0.05 - (pre and post treatment of NC) NS	(mechanical and cold allodynia) - BVA may attenuate oxaliplatin-induced neuropathic pain by altering the action potential threshold in A-fibre dorsal root ganglia neurons.	Not reported	[43]
Mechanical allodynia	Male Sprague Dawley rats (200–250 g), sciatic nerve CCI induced mechanical allodynia (*n* = 10)	BV (*A. mellifera*)	- E: CCI surgery rats with BV injected subcutaneously twice a day for two consecutive weeks (AR: ST36 acupoint, dose: 0.25 mg/kg, BV dissolved in a volume of 50 μL saline) - NC: CCI surgery rats with saline injected subcutaneously twice a day for two consecutive weeks (AR: ST36 acupoint, dose: not reported)	Mechanical allodynia (von Frey test) - (E vs. NC) NS (after 1 day), *p* < 0.01 (after 4 days), and *p* < 0.001 (after 7, 10, 13 days)	- BV treatment associated with the activation of the LC noradrenergic system and with a reduction in spinal pNR1	Not reported	[44]
Mechanical allodynia	Male Sprague Dawley rats (180–200 g), laminectomy was performed between the T11 to T12 vertebral segments (*n* = 8)	BV (*A. mellifera*)	- E: spinal cord injury rats with BV injected subcutaneously twice a day from 15 to 20 days post-surgery (AR: ST36 acupoint, dose: 0.25 mg/kg, BV dissolved in a volume of 50 μL saline) - NC: spinal cord injury rats with saline injected subcutaneously twice a day from 15 to 20 days post-surgery (AR: ST36 acupoint, dose: not reported)	Mechanical allodynia (ipsilateral paw) - (E vs. NC) NS (after 1 day), *p* < 0.05 (after 3 days), *p* < 0.05 (after 5 days), NS (after 7 days), NS (after 10 day), and NS (after 14 day)	- Reduction in mechanical allodynia is mediated by the suppression of spinal astrocyte or microglia activation	Not reported	[45]
Mechanical and cold allodynia	Male Sprague-Dawley rats (190–210 g), CIPN model was established using daily vincristine infusions (E1, *n* = 12/E2, *n* = 12/E3, *n* = 12)	BV (*A. mellifera*), PLA_2_ (*A. mellifera*), melittin (*A. mellifera*)	- E1: vincristine-induced rats with BV subcutaneously injected once (AR: ST36 acupoint, dose: 1.0 mg/kg, BV dissolved in a volume of 50 μL saline) - E2: vincristine-induced rats with melittin subcutaneously injected once (AR: ST36 acupoint, dose: 0.5 mg/kg, melittin dissolved in a volume of 50 μL saline) - E3: vincristine-induced rats with PLA_2_ (0.12 mg/kg) subcutaneously injected once (AR: ST36 acupoint, dose: 0.12 mg/kg, PLA_2_ dissolved in a volume of 50 μL saline) - NC: vincristine-induced rats with mice with saline subcutaneously injected once (AR: ST36 acupoint, dose: not reported)	Mechanical allodynia (von Frey test) - (E1 vs. NC) *p* < 0.01 (after 30 min), *p* < 0.05 (after 60 min), and NS (after 120 min) - (E2 vs. NC) *p* < 0.01 (after 30 min), and *p* < 0.05 (after 60, 120 min) - (E3 vs. NC) NS (after 30, 60, 120 min) Cold allodynia (acetone test) - (E1 vs. NC) *p* < 0.05 (after 30 min), and *p* < 0.01 (after 60 min) - (E2 vs. NC) NS (after 30, 60, 120 min) - (E3 vs. NC) NS (after 30, 60, 120 min)	(mechanical and cold allodynia) - BVA manoeuvre attenuated the hyperexcitation of spinal WDR neurons in rats with neuropathy - BVA-induced analgesia was mediated by the descending noradrenergic pathway, which mainly originates from the LC	Not reported	[46]
Cold allodynia	Male Sprague Dawley rats (200–220 g), oxaliplatin induced cold allodynia (E, *n* = 8/AC, *n* = 5)	BV (*A. mellifera*)	- E: oxaliplatin-induced rats with DL-pchlorophenylalanine (AR: intraperitoneal route, dose 150 mg/kg) daily injected for three days and BV injected once (AR: GV3 acupoint, dose: 0.25 mg/kg, BV dissolved in a volume of 0.05 cc saline) - AC: oxaliplatin-induced rats with saline (AR: GV3 acupoint, volume: 0.05 cc) and BV injected once (AR: GV3 acupoint, dose: 0.25 mg/kg, BV dissolved in a volume of 0.05 cc saline)	Cold allodynia (acetone test) - (pre and post treatment of E) NC - (pre and post treatment of AC) *p* < 0.001	- spinal 5-HT3 receptors are activated to exert the anti-allodynic effect of BVA	Not reported	[47]
Cold allodynia	Male Sprague-Dawley rats (average 200 g), oxaliplatin induced cold allodynia (E, *n* = 7/AC1, *n* = 8/AC2, *n* = 6)	BV (*A. mellifera*)	- E: oxaliplatin-induced rats with BV subcutaneously injected once (AR: GV3 acupoint, dose: 0.25 mg/kg, BV dissolved in saline at concentrations of 0.025 mg/mL) - AC1: E group + mecamylamine administered once (AR: intraperitoneal route, dose: 2 mg/kg) - AC2: E group + atropine administered once (AR: intraperitoneal route, dose: 1 mg/kg)	Cold allodynia (acetone test) - (post injection of E) *p* < 0.05 (after 30 min), and *p* < 0.01 (after 60 min) - (post injection of AC1) *p* < 0.001 (after 30, 60 min) - (post injection of AC2) NS (after 30, 60 min)	- BVA treatment alleviates oxaliplatin induced neuropathic cold allodynia in rats via the activation of nicotinic acetylcholine receptors, especially spinal α4β2 receptors.	Not reported	[48]
Cold allodynia	Male Sprague-Dawley rats (210–250 g), oxaliplatin induced cold allodynia (E1, *n* = 4/E2, *n* = 4/E3, *n* = 4)	BV (*A. mellifera*)	- E: oxaliplatin-induced rats with BV subcutaneously injected once (AR: GV3 acupoint, dose: 1.0 mg/kg, BV dissolved in a volume of 0.05 cc saline) - AC1: oxaliplatin-induced rats with BV subcutaneously injected once (AR: LI11 acupoint, dose: 1.0 mg/kg, BV dissolved in a volume of 0.05 cc saline) - AC2: oxaliplatin-induced rats with BV subcutaneously injected once (AR: ST36 acupoint, dose: 1.0 mg/kg, BV dissolved in a volume of 0.05 cc saline)	Cold allodynia (acetone test) - (pre and post treatment of E) *p* < 0.05 (after 1 h), and *p* < 0.001 (after 2 h) - (pre and post treatment of AC1) *p* < 0.05 (after 1 h), and NS (after 2 h) - (pre and post treatment of AC2) *p* < 0.05 (after 1 h), and NS (after 2 h)	- BVA alleviates oxaliplatin induced cold allodynia in rats, at least partly, through activation of the noradrenergic system.	Not reported	[49]
Cold allodynia	Male Sprague Dawley rats (180–230 g), CCI induced cold allodynia (E1, *n* = 6/E2, *n* = 6)	BV (*A. mellifera*)	- E1: CCI surgery rats with BV injected subcutaneously twice a day for 2 weeks (AR: ST36 acupoint, dose: 0.25 mg/kg, BV dissolved in a volume of 50 μL saline) - E2: CCI surgery rats with BV injected subcutaneously, twice a day for 2 weeks (AR: ST36 acupoint, dose: 2.5 mg/kg, BV dissolved in a volume of 50 μL saline) - NC: CCI surgery rats with saline injected subcutaneously, twice a day for 2 weeks (AR: ST36 acupoint, dose: not reported)	Cold allodynia (acetone test) - (E1 vs. NC) NS (after 15, 30, 45, 60, 90, 120 min) - (E2 vs. NC) *p* < 0.01 (after 45, 90 min), and *p* < 0.001 (after 60 min)	- BV treatment could relieve cold allodynia via activation of spinal α2-adrenoceptors	Not reported	[50]
Intervertebral disc disease induced neurological pain	Canines (both sexes), intervertebral disc disease induced neurological pain (*n* = 17)	BV (*A. mellifera*)	- E: AC group + intervertebral disc disease induced canines with BV subcutaneously injected twice a week for 6 weeks (AR: LI4, SI3, KI3, ST36, BL23, BL40, GB30, GB34, LR3, GV1, Baihui, and Ashi acupoints, dose: 5.0 mg/kg BV dissolved in a volume of 50 μL saline injected 0.1 mL for each acupoint) - AC: intervertebral disc disease induced canines with oral prednisone (1 mg/kg) together with the NSAID carprofen (2.2 mg/kg) for 7 days. Ranitidine (2 mg/kg) for 5 or 7 days was administered to prevent gastrointestinal disturbance	Neurological pain (myelopathy scoring system) - (E vs. AC) *p* = 0.245 (all group), *p* = 0.59 (mild group), *p* = 0.001 (moderate group), and *p* = 0.014 (severe group)	Not reported	Not reported	[51]

Abbreviations: *A. mellifera: Apis* *mellifera*, AC: active control group, AR: administration route, BV: bee venom, BVA: bee venom acupuncture, CCI: chronic constrictive injury, CIPN: chemotherapy induced peripheral neuropathic pain, DRG: dorsal root ganglia, E: experimental group, IENFs: intraepidermal nerve fibres, LC: locus coeruleus, NC: normal control group, NS: no significant effects, NSAID: non-steroidal anti-inflammatory drugs, PLA_2_: phospholipase A_2_, PBS: phosphate-buffered saline, SNL: spinal nerve ligation, TRPA1: transient receptor potential ankyrin type 1, TRPM8: transient receptor potential melastatin type 8, TRPV1: transient receptor potential vanilloid type 1, WDR: wide dynamic range.

**Table 6:** Experimental studies on therapeutic application of bee venom for post-ischaemic pain and prostatic pain.

Disease	Study Design (Number of Patient or Animal Treated by BV)	Venom/Compound/(Bee Species)	Intervention (Dilution Ratio, Amount of Bee Venom Use)	Main Results	Mechanism/Molecular Response	Adverse Events of BVA	Reference
Chronic post- ischaemic pain	Male C57/Bl6 mice (25–30 g), chronic post-ischaemic pain model was induced in mice with isoflurane by placing a tight-fitting O-ring with a 5/64-inch internal diameter around the left ankle for 3 h (*n* = 18)	BV (*A. mellifera*)	- E1: chronic post-ischaemic pain model mice with BV injected (AR: dorsum of the ipsilateral hind paw, dose: 1.0 mg/kg BV injected once) - E2: chronic post-ischaemic pain model mice with BV injected (AR: dorsum of the ipsilateral hind paw, dose: 1.0 mg/kg BV injected twice) - E3: chronic post-ischaemic pain model mice with BV injected (AR: dorsum of the ipsilateral hind paw, dose: 1.0 mg/kg BV injected 3 times) - NC: chronic post-ischaemic pain model mice with saline injected once (AR: dorsum of the ipsilateral hind paw, dose: same dose of E1 group)	Mechanical allodynia (von Frey test) - (E1 vs. NC) *p* < 0.05 (after 30 min), *p* < 0.05 (after 60 min), *p* < 0.05 (after 90 min), NS (after 120 min), NS (after 180 min), NS (after 240 min), and NS (after 24 h) - (E2 vs. NC) *p* < 0.05 (after 30 min), *p* < 0.05 (after 60 min), *p* < 0.05 (after 90 min), *p* < 0.05 (after 120 min), NS (after 180 min), NS (after 240 min), and NS (after 24 h) - (E3 vs. NC) *p* < 0.05 (after 30 min), *p* < 0.05 (after 60 min), *p* < 0.05 (after 90 min), *p* < 0.05 (after 120 min), *p* < 0.05 (after 180 min), NS (after 240 min), and NS (after 24 h)	- Antiallodynic effect was objectively proven through a reduction in the increased expression of NK-1 receptors in dorsal root ganglia.	No adverse events	[52]
Chronic prostatic pain	Male Sprague-Dawley rats (400–450 g), complete Freund’s adjuvant induced chronic prostatic pain (*n* = 15)	Melittin (*A. mellifera*)	- E: complete Freund’s adjuvant induced rats with melittin injected into both right and left lobes of the prostate once (dose: 0.05 mg melittin in a volume of 0.2 mL saline) - NC: rats with sham operation - AC: complete Freund’s adjuvant induced rats with saline injected into both right and left lobes of the prostate once (volume: 0.2 mL)	Chronic prostatic pain (Threshold of mechanical pain) - (E vs. NC) NS (after 0 day), NS (after 6 days), *p* < 0.01 (after 12 days), and *p* < 0.01 (after 18 days) - (E vs. AC) NS (after 0 day), NS (after 6 days), NS (after 12 days), and *p* < 0.05 (after 18 days)	-Melittin could alleviate complete Freund’s adjuvant induced prostatitis pain in rat by suppressing cyclooxygenase-2 expression	Not reported	[53]

Abbreviations: *A. mellifera*: *Apis mellifera*, AC: active control group, AR: administration route, BV: bee venom, BVA: bee venom acupuncture, E: experimental group, NC: normal control group, NK-1: neurokinin type 1, NS: no significant effects.

## Data Availability

The data will be made available upon reasonable request.
